# Ossifying Subperiosteal Hematoma Caused by a Plexiform Neurofibroma

**DOI:** 10.5334/jbsr.2186

**Published:** 2020-11-06

**Authors:** Steven Van den Berge, Hugo Declercq, Steven Pans

**Affiliations:** 1 UZ Leuven, BE; 2 A.Z. Sint-Blasius Dendermonde, BE; 3 University Hosptals Leuven, BE

**Keywords:** neurofibromatosis type 1, plexiform neurofibroma, subperiosteal hematoma

## Abstract

**Teaching Point:** Subperiosteal haemorrhage is a rare complication of a plexiform neurofibroma which may mimic a malignant peripheral nerve sheath tumour.

## Case Report

A 15-year-old male was referred by his general practitioner for evaluation of non-traumatic, painful lump on the medial aspect of his right tibia. The patient was known with neurofibromatosis type 1 (NF-1).

**Figure 1:**
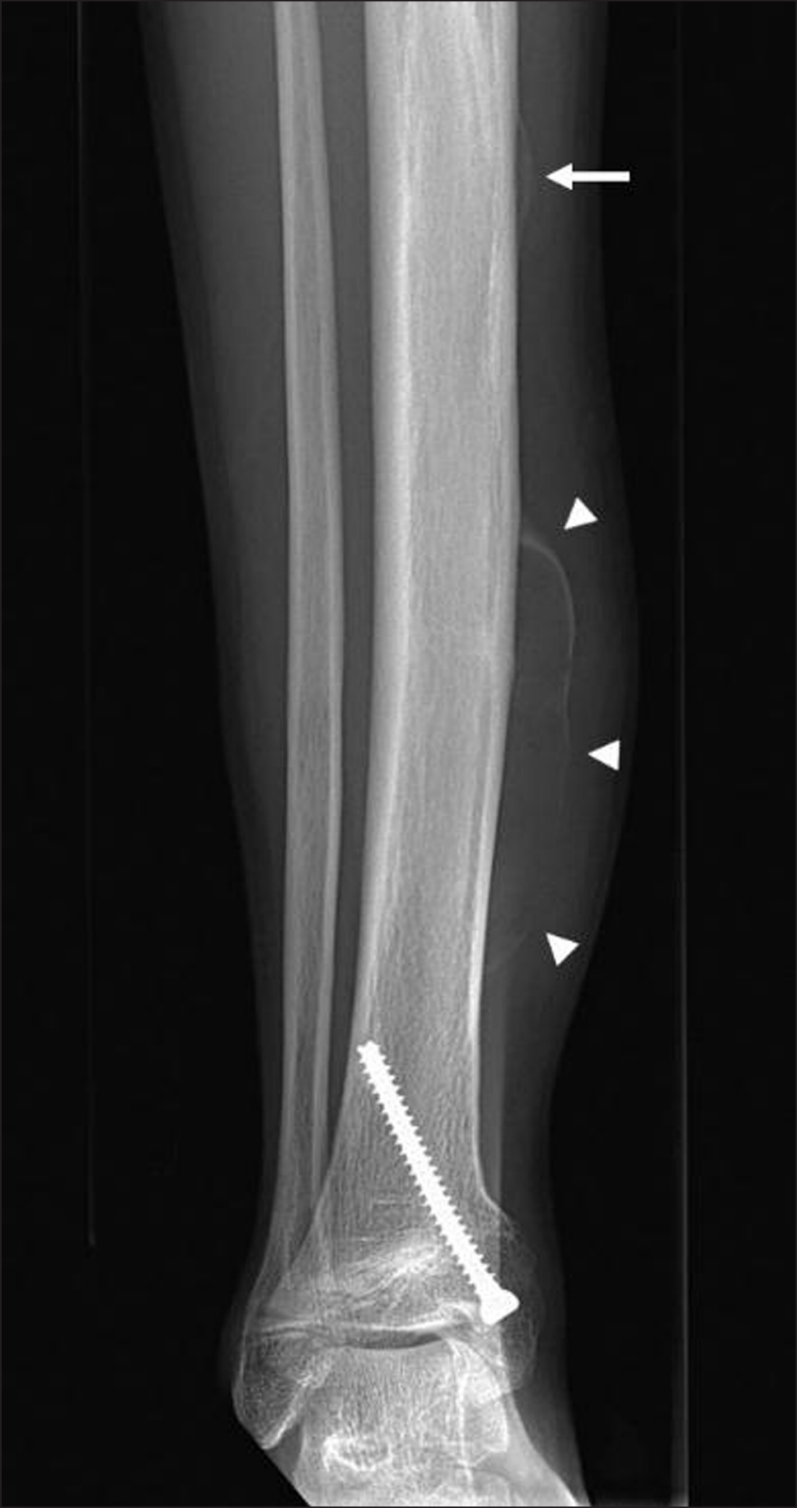


**Figure 2:**
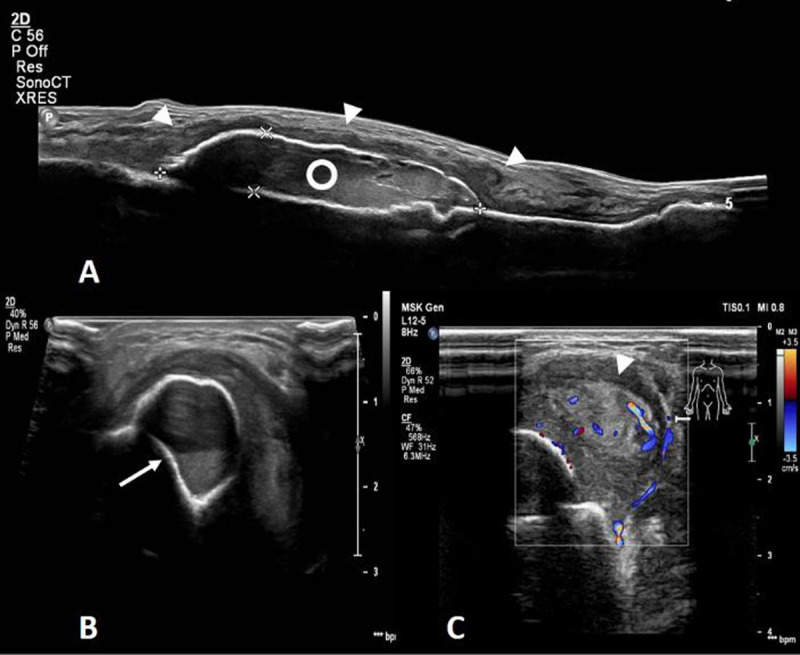


**Figure 3:**
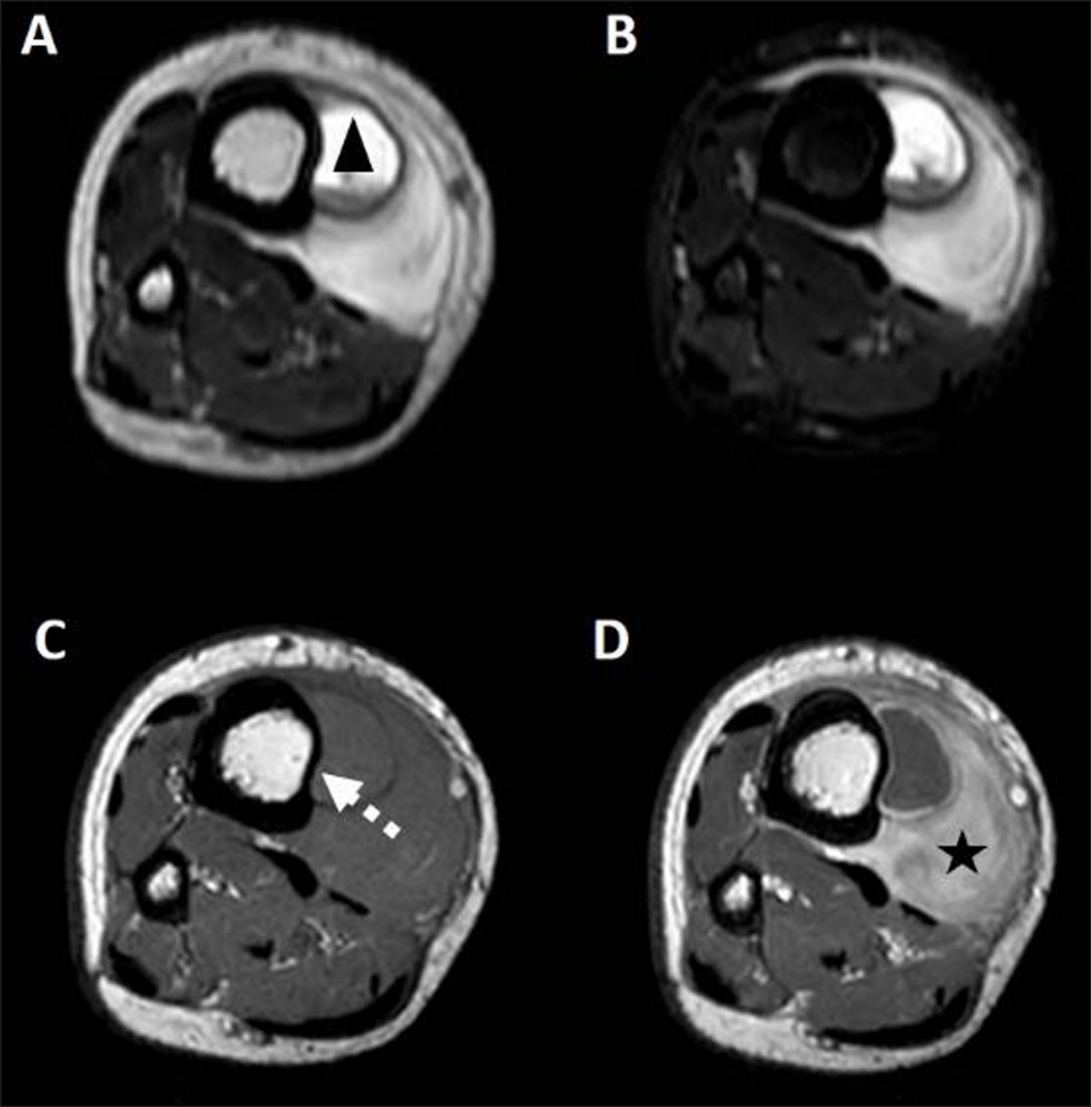


Radiographs (Figure 1) demonstrated a soft tissue swelling on the medial side of the tibial shaft outlined by a thin calcified rim (arrowheads). A smaller, similar lesion could be seen more cranially (arrow). Ultrasound (US) (Figure 2A-C) showed a subperiosteal collection (circle) with an internal fluid-fluid level (arrow) as well as calcification of the surrounding periosteum. The calcified periosteum is surrounded by a heterogeneous, vascularized soft tissue cuff (arrowheads). MRI (Figure 3A-D) demonstrated a subperiosteal collection with signal properties of a chronic hematoma (triangle): T1/T2-isointense in the periphery of the lesion but T1-iso/T2-hyperintense in the centre (Figure 3C and 3A, respectively, for T1- and T2-weighted imaging). Moreover, there was the additional finding of a T2-hyperintense, T1-isointense, fat-poor (Figure 3B), enhancing tissue (star in Figure 3D), located medially and posteriorly with respect to the hematoma, corresponding to the described soft tissue cuff on US. These MRI features were suggestive of a plexiform neurofibroma of the saphenous nerve, that has caused an ossifying subperiosteal hematoma due to chronic erosion of the tibial cortex. Note the smooth cortical thinning on MRI (dashed arrow), the mass effect exerted on the tibialis posterior and flexor digitorum muscles as well as the surrounding soft tissue oedema.

After three weeks, the swelling and pain had decreased significantly and it was decided not to perform any more imaging.

## Comment

Although osseous manifestations of NF-1 are not uncommon, the occurrence of ossifying subperiosteal hematomas due to direct periosteal involvement by a plexiform neurofibroma is exceedingly rare [1].

The pathogenesis of such hematoma, traditionally termed “subperiosteal bone cyst”, is thought to be due to a combination of vascular fragility and weakened periosteal attachment. One should consider abscess, subperiosteal aneurysmal bone cyst, and subperiosteal vascular malformation in the differential diagnosis. Timing of the appearance of the calcified periosteal elevation or “eggshell” aspect is not clear, with some reports as short as 5–7 days. Later on, the hematoma may undergo resorption or complete ossification [1].

NF-1 patients have a 2–10% lifetime risk of malignant transformation of a neurofibroma into a malignant peripheral nerve sheath tumour (MPNST). Certain MRI findings suggestive of malignancy (e.g. perilesional oedema) are difficult to apply in cases with concurrent subperiosteal haemorrhage because they can be inherent to the bleeding. As a result, differentiating an MPNST from a plexiform neurofibroma complicated with subperiosteal cyst formation poses a radiological challenge. The latter is especially true when the neurofibroma is located (sub)periosteal [1].

Watchful waiting with follow-up radiograph and MRI 3–4 weeks after the initial presentation is recommended. At this time, the lesion should be scrutinized for changes, especially more erratic enhancement, which could suggest malignant degeneration. If the lesion has significantly enlarged without concurrently increased haemorrhage, then biopsy and/or complete surgical resection should be contemplated.
